# Differentiating the Oxidation States of Copper in a Type-I Copper Protein Using XANES Spectra in the Crystalline State: A Comparison with Single-Crystal X-ray Crystallography Study

**DOI:** 10.1063/4.0001153

**Published:** 2025-10-27

**Authors:** Narayanasami Sukumar, Sahana L Sukumar, George E Sterbinsky

**Affiliations:** 1 Cornell University, Argonne, Illinois, USA; 2 Loyola University, Chicago, Illinois, USA; 3 Argonne National Laboratory, Argonne, Illinois, USA

## Abstract

Understanding the oxidation states of transition metals in metalloproteins is critical for elucidating their biochemical functions. Among the available spectroscopic techniques, X-ray Absorption Near Edge Structure (XANES) spectroscopy offers a powerful, element-specific method for probing the electronic environment and oxidation state of metal centers in biological macromolecules. The shape, position, and intensity of the absorption edge in a XANES spectrum can provide direct insights into the valence state and coordination geometry of the metal ion, making it a particularly valuable tool for studying redox-active metalloproteins under near-physiological conditions.

In this study, we apply XANES spectroscopy to investigate the copper oxidation states in amicyanin, a well- characterized Type-I copper protein, in its crystalline form. Type-I copper sites are highly conserved among various redox proteins found in bacteria, plants, and animals, and are known for their intense blue color due to strong ligand-to-metal charge transfer transitions. These proteins serve as efficient electron transfer (ET) agents, playing vital roles in biological processes such as respiration and metabolism.

Amicyanin, a representative member of the cupredoxins family, features a single copper ion coordinated by a distorted tetrahedral geometry with **three strong equatorial ligands** (a cysteine and two histidines) and one weak axial ligand, typically methionine. It functions as an electron acceptor for the enzyme methylamine dehydrogenase and transfers it to cytochrome C551i.

Our objective is to acquire XANES spectra from single crystals of amicyanin in both **oxidized and reduced states**. These spectra will be analyzed to determine the oxidation state of the copper center and to detect any associated changes in the local electronic environment. Results from XANES will be compared with structure from single-crystal X-ray crystallography, which provides complementary structural information about the copper coordination sphere.

This comparative study aims to deepen our understanding of how changes in oxidation state correlate with local structural features at the active site, enhancing our ability to interpret redox mechanisms in copper proteins at atomic resolution. Results from these complementary techniques will be presented**,** highlighting how their synergy enables a deeper insight into structure-function relationships in metalloproteins.

